# Mental Health Interventions in Refractory Chronic Spontaneous Urticaria: A Call to Expand Treatment Guidelines

**DOI:** 10.7759/cureus.81443

**Published:** 2025-03-29

**Authors:** George N Konstantinou, Indrashis Podder, Gerasimos Konstantinou

**Affiliations:** 1 Allergy and Clinical Immunology, 424 General Military Training Hospital, Thessaloniki, GRC; 2 Dermatology and Venereology, College of Medicine and Sagore Dutta Hospital, Kolkata, IND; 3 Psychiatry, University of Toronto, Toronto, CAN; 4 Centre for Addiction and Mental Health, University of Toronto, Toronto, CAN; 5 Centre for Mental Health, University Health Network, Toronto, CAN

**Keywords:** anxiety, chronic spontaneous urticaria, cognitive behavioral therapy, corticosteroids, cyclosporin, escitalopram, mental health, neuroimmune, quality of life, stress

## Abstract

Chronic spontaneous urticaria (CSU) is a complex inflammatory skin condition that severely impacts patients’ quality of life. For some patients, conventional treatments, including second-generation antihistamines, omalizumab, and cyclosporine A, fail to achieve sustained control. Emerging evidence suggests that psychiatric comorbidities, such as generalized anxiety disorder (GAD), exacerbate CSU through neuro-immuno-cutaneous (the interaction between the nervous system, immune system, and skin) mechanisms. We present the case of a 35-year-old female with refractory CSU and GAD. Despite escalating doses of omalizumab and the addition of cyclosporine A, disease control remained unstable. The introduction of cognitive behavioral therapy (CBT) and escitalopram resulted in significant improvement, achieving complete resolution of symptoms within eight weeks. Following discontinuation of both escitalopram and omalizumab, urticaria relapsed after a stressful event and during pregnancy. The reintroduction of escitalopram and CBT reestablished full control of urticaria. The patient continues on escitalopram and CBT without needing additional pharmacological intervention for CSU. This case underscores the importance of incorporating mental health interventions in managing refractory CSU, as psychiatric comorbidities may exacerbate CSU by intensifying neuro-immune interactions, particularly during stressful periods. Integrating mental health care into treatment guidelines can offer significant benefits, improving symptom control, reducing the need for aggressive pharmacotherapy, and enhancing patients’ quality of life.

## Introduction

Chronic spontaneous urticaria (CSU) is a relatively common inflammatory skin disorder characterized by recurrent pruritic wheals with or without angioedema lasting at least six weeks without an identifiable provoking factor. Epidemiological studies indicate that CSU affects approximately 0.5-1% of the general population, contributing to a significant burden on healthcare systems and patients’ quality of life. The term CSU characterizes an entity that arises spontaneously. However, potential aggravating factors such as drugs (with non-steroidal anti-inflammatory drugs (NSAIDs) being a common trigger), foods (including preservatives), hormonal fluctuations (including menstruation), and infections may further contribute to symptom exacerbation [1]. Persistent symptoms and unpredictable flare-ups often result in physical discomfort and psychological distress [2,3], creating a vicious cycle where stress further exacerbates disease activity [4].

Standard therapeutic guidelines recommend second-generation antihistamines as first-line treatment, with omalizumab and cyclosporine A (CsA) indicated for antihistamine-resistant cases. Despite these interventions, a significant proportion of CSU patients remain refractory [5], underscoring the need for alternative therapeutic approaches. Recent evidence highlights the frequent coexistence of psychiatric comorbidities, notably anxiety and depression, involving complex interactions between neuropeptides, cytokines, and dysregulation of the hypothalamic-pituitary-adrenal (HPA) axis [4,6-8].

Clinicians routinely assess CSU severity and treatment responses using validated scoring tools. The Urticaria Control Test (UCT) evaluates disease control from a patient's perspective over four weeks, with scores below 12 indicating poor disease control [9]. The Urticaria Activity Score over seven days (UAS7) quantifies wheal formation and itch intensity, ranging from 0 (no disease activity) to 42 (severe activity) [10,11]. The Dermatology Life Quality Index (DLQI) assesses the overall impact of skin diseases on patient quality of life, with scores above 5 indicating moderate-to-severe impairment [12].

Although these scoring systems effectively measure CSU severity, they do not directly address the substantial psychiatric burden frequently observed in these patients. Given the high prevalence of psychiatric comorbidities, such as anxiety and depression, and the fact that many CSU patients remain refractory even to advanced therapies like omalizumab, integrating mental health assessments and interventions into CSU treatment may improve symptom management, reduce disease severity, and enhance patient outcomes. The case reported below illustrates the potential benefits of such an integrated approach. The patient provided informed consent for this case report publication, and all the information that might identify her has been excluded to ensure confidentiality.

## Case presentation

A 35-year-old female with CSU was initially presented in March 2014 with an episode of acute urticaria with angioedema. She demonstrated a partial response to levocetirizine 5 mg once daily but achieved better symptom control with ebastine 20 mg once daily. Despite antihistamine treatment, symptoms persisted beyond six weeks, meeting the criteria for CSU. At that time, she had a UCT score of <12, a UAS7 ≥3, and a DLQI >5, indicating a significant quality-of-life impact. This episode lasted eight months.

In March 2017, during febrile gastroenteritis, another urticaria episode occurred, which progressed to CSU as symptoms persisted for more than six weeks. At this point, she was referred to our department for an allergological evaluation for the first time. All physical examinations and laboratory tests, including differential blood count (to assess for any hematologic abnormalities), C-reactive protein (to detect systemic inflammation), thyroid-stimulating hormone (to evaluate thyroid function, as thyroid disorders can be associated with chronic urticaria), anti-thyroid peroxidase (anti-TPO) and anti-thyroglobulin (anti-TG) antibodies (to screen for autoimmune thyroid disease), and stool examination for parasites (to rule out parasitic infections as potential triggers), were unremarkable. Total IgE was 105 IU/mL. The patient had no history of significant illnesses, no known allergies, and no history of surgeries. Apart from antihistamines, she was not taking any medications or supplements at this initial assessment. Her vital signs were normal, and her overall appearance was that of a healthy individual aside from the characteristic urticaria rash. She had no family history of allergies or urticaria.

Despite escalating treatment to high-dose ebastine at 20 mg twice daily, symptom control remained inadequate. Therefore, CsA at a dose of 3 mg/kg was initiated in May 2017, following then-current guidelines for antihistamine-resistant CSU [13]. Due to a lack of clinical improvement, CsA was discontinued four weeks later, and omalizumab 300 mg every four weeks (Q4W) was started, resulting in significant clinical improvement within eight weeks of therapy, as reflected by a DLQI of 3, UCT of 13, and UAS7 of 1.

Over the next year, she had several CSU exacerbations, frequently perimenstrual or during periods of stress. Treatment escalation by combining CsA with omalizumab resulted in drug-induced hirsutism, leading to the discontinuation of CsA. At this point, additional investigations were performed to identify potential underlying causes, including serum tryptase (to assess mast cell activation syndromes), antinuclear antibodies (ANA) and rheumatoid factor (RF) (to screen for systemic autoimmune diseases), stool examination for Helicobacter pylori (given its association with chronic urticaria), antibodies for hepatitis A, hepatitis B, hepatitis C, and HIV (to detect viral infections that could exacerbate urticaria), gynecological examination (to identify any gynecological infections or conditions), dental examination (to detect dental infections or issues), and complement studies (C3, C4, and CH50) (to evaluate for complement pathway abnormalities). All results were within normal limits. As a last option, the omalizumab dose was gradually increased to 450 mg Q4W by October 2018 and further escalated to 600 mg Q4W in June 2019, achieving stable disease control (DLQI: 2, UCT: 14, UAS7: 0). However, during the COVID-19 pandemic, CSU control was lost with severe symptoms associated with increased anxiety. Partial symptom control was achieved through higher doses of omalizumab (up to 600 mg every two weeks (Q2W)) combined with oral corticosteroids.

In June 2021, during the COVID-19 lockdowns, the patient was diagnosed with generalized anxiety disorder (GAD). Treatment was initiated with cognitive behavioral therapy (CBT) and escitalopram, a selective serotonin reuptake inhibitor and first-line pharmacological treatment for GAD, at a dose of 20 mg once daily, which was well tolerated. Astonishingly, the urticarial symptoms completely resolved within eight weeks with DLQI: 1, UCT: 16, and UAS7: 0 for the first time after CSU onset. The patient was able to discontinue antihistamines after nearly five years but continued omalizumab at 300 mg Q4W.

On October 10, 2022, the patient remained controlled (DLQI: 1, UCT: 16, UAS7: 0), and consequently, omalizumab was discontinued. One month post-discontinuation, the patient had no symptoms at all, and it was decided to stop both escitalopram and CBT. However, 50 days later, following a significantly stressful event, a refractor to both antihistamines and corticosteroids, urticaria relapsed. The patient resumed escitalopram and CBT under psychiatric supervision, and urticaria control was gradually reestablished within two months, having DLQI: 0, UCT: 16, and UAS7: 0 in March 2023.

In February 2024, upon confirmed pregnancy, escitalopram was discontinued at the patient’s request. Within 10 days, urticaria relapsed but was partially managed with cetirizine 10 mg daily and methylprednisolone 8 mg daily. Unfortunately, the pregnancy ended in miscarriage four weeks later. Subsequently, escitalopram was reintroduced, resulting in full urticaria control within six weeks (UCT: 16, UAS7: 0).

As of March 2025, the patient continues on escitalopram and CBT, maintaining sustained control of CSU, without the need for antihistamines or corticosteroids.

## Discussion

This case highlights the challenges in managing refractory CSU, particularly in patients with psychiatric comorbidities such as GAD. To our knowledge, this is the first reported patient demonstrating substantial improvement of refractory CSU symptoms following combined psychiatric interventions, including cognitive behavioral therapy and escitalopram.

Conventional pharmacological treatments, including high-dose antihistamines, CsA, and escalated omalizumab dosing, failed to provide long-term control for this patient. Omalizumab, administered at 300 mg every four weeks, has demonstrated response rates ranging from 65% to 85%. Several studies have explored the efficacy of higher doses in patients unresponsive to the standard regimen [1]. A recent real-life study reported that increasing the dose up to 600 mg every two weeks improved response rates, with 78% of patients achieving responder status, compared to 70% on the standard dosing schedule [14]. However, it is important to note that such omalizumab up-dosing is off-label. In our case, the patient initially responded to omalizumab but later experienced relapses despite increasing doses, highlighting the need for individualized management approaches.

The frequent symptom exacerbations (Table 1), often associated with elevated stress levels, underscore the potential influence of neuro-immune interactions on CSU pathology [4,8]. The interplay between chronic urticaria and psychological stress is increasingly recognized through the prism of psycho-neuro-immunology. Stress activates neuroendocrine pathways such as the hypothalamic-pituitary-adrenal axis and the sympathetic-adrenal-medullary axis, leading to alterations in cortisol release and heightened sympathetic activity, which can exacerbate skin inflammation by influencing mast cell activation, neuropeptide release, and immune dysregulation. In particular, neuropeptides like substance P and neurotrophic growth factor released in response to stress have been implicated in mast cell activation, cytokine production, and increased inflammation characteristic of urticarial lesions. Psychological stressors appear to intensify these processes and thereby increase disease severity [4,8,15]. Additionally, hormonal fluctuations occurring during pregnancy and following miscarriage may modulate mast cell activation and immune responses, potentially contributing to exacerbations of CSU in predisposed individuals [16]. However, further studies are necessary to clarify these interactions, which may not be fully addressed by omalizumab's mechanism of action, which primarily targets IgE-mediated pathways. Consequently, patients with such dysregulated pathways may require alternative or adjunctive therapies to achieve optimal disease control. In this case, the incorporation of CBT and escitalopram led to significant and sustained clinical improvement, likely by mitigating the stress-induced immune dysregulation. Selective serotonin reuptake inhibitors (SSRIs) such as escitalopram may modulate CSU symptoms via several molecular mechanisms, although these pathways remain incompletely understood. Sustained administration of SSRIs leads to serotonin depletion in platelets, potentially reducing their activation and subsequent inflammatory responses implicated in CSU pathogenesis [17]. Additionally, SSRIs have immunosuppressive properties that include reducing lymphocyte proliferation and modulating cytokine secretion, which could diminish inflammatory cascades involved in CSU [18]. Escitalopram may also influence substance P levels, a neuropeptide known to be elevated in CSU patients with comorbid depression, thereby potentially reducing mast cell activation and skin inflammation [19]. However, it is important to note that direct evidence linking these mechanisms specifically to CSU response is limited, and further research is needed to elucidate these potential molecular pathways.

**Table 1:** Timeline of Clinical Symptoms, Treatments, and Responses UCT: urticaria control test; UAS: Urticaria Activity Score; DLQI: Dermatology Life Quality Index; GAD: generalized anxiety disorder; CBT: cognitive behavioral therapy; CSU: chronic spontaneous urticaria; QD: everyday; Q4 weeks: every four weeks UCT (≥12 = controlled, <12 = uncontrolled); UAS7 (≤6 = well-controlled, 7–27 = moderate, ≥28 = severe); DLQI (>5 = moderate-to-severe impairment)

Date	Event	Treatment	Outcome
March 2014	Initial onset of urticaria with angioedema.	Levocetirizine 5 mg qd, ebastine 20 mg qd.	Partial response to levocetirizine; better control with ebastine.
March 2017	Acute urticaria episode following febrile gastroenteritis.	High-dose ebastine 40 mg QD.	Symptoms persisted.
May 2017	Initiation of cyclosporine.	Cyclosporine 3 mg/kg.	Inadequate response; cyclosporine discontinued after four weeks.
June 2017	Start of omalizumab therapy.	Omalizumab 300 mg q4 weeks.	Significant improvement within eight weeks (DLQI: 3, UCT: 13, UAS: 1).
October 2018	Exacerbation linked to stress and menstrual cycle.	The omalizumab dose was increased to 450 mg.	Partial control was achieved.
June 2019	Further exacerbations.	The omalizumab dose was further increased to 600 mg.	Stable control was achieved (DLQI: 2, UCT: 14, UAS: 0).
COVID-19 Era	Disease relapse attributed to heightened anxiety during lockdowns.	Increased omalizumab dose; corticosteroids added.	Partial relief.
June 2021	Diagnosis of GAD.	Escitalopram 20 mg QD and CBT initiated.	Full resolution of symptoms after eight weeks (DLQI: 1, UCT: 16, UAS7: 0). Antihistamines discontinued.
October 2022	Discontinuation of omalizumab.	Omalizumab discontinued.	Remained well-controlled for one month (DLQI: 1, UCT: 16, UAS: 0).
December 2022	Discontinuation of escitalopram and CBT.	No medication for CSU.	
January 2023	Stress-inducing event leading to urticaria relapse.	Escitalopram and CBT resumed.	Control was restored within two months (March 2023).
February 2024	Pregnancy; escitalopram discontinued.	Cetirizine 10 mg QD and methylprednisolone 8 mg QD for seven days.	Urticaria relapsed within 10 days; partial symptom control was achieved.
March 2024	Miscarriage	Escitalopram reintroduced.	Disease control was restored within six weeks (UCT: 16, UAS7: 0).
As of March 2025	The patient continues maintenance therapy.	Escitalopram 20 mg qd and CBT.	Sustained symptom control to date.

Psychiatric comorbidities seem to be common among patients with chronic urticaria, with a prevalence as high as 31.6% [6,7]. For patients unresponsive to standard CSU therapies, mental health strategies, including pharmacotherapy (e.g., escitalopram) and psychotherapy (e.g., CBT), can provide improved symptom management. These findings support the integration of mental health care into CSU treatment guidelines, promoting a more holistic approach to patient management. By addressing both the physical and psychological aspects of CSU, clinicians can potentially reduce disease severity and exacerbations, minimize reliance on intensive pharmacological interventions, and improve quality of life. Given the high prevalence of psychiatric comorbidities among CSU patients and the subset that remains refractory even to advanced therapies like omalizumab, mental health evaluations, and interventions should be considered as a critical next step in their diagnostic and therapeutic algorithm.

## Conclusions

This case highlights the importance of identifying and managing psychiatric comorbidities in refractory chronic spontaneous urticaria. Conventional therapies, even at escalated doses, may fail in patients with significant psychological stress or underlying mental health disorders. The remarkable response to cognitive behavioral therapy and escitalopram in this patient illustrates the potential of mental health interventions as an essential component of CSU management. This case illustrates the potential benefits of incorporating psychological care into current CSU treatment guidelines, suggesting that addressing stress-induced immune dysregulation may contribute to reducing disease severity and improving long-term outcomes. By adopting a holistic approach that addresses both physical and psychological aspects of the disease, clinicians can enhance the quality of life and reduce the need for prolonged or intensive pharmacological interventions.
